# In silico design and molecular docking study of CDK2 inhibitors with potent cytotoxic activity against HCT116 colorectal cancer cell line

**DOI:** 10.1186/s43141-020-00066-2

**Published:** 2020-09-15

**Authors:** Fabian Adakole Ikwu, Yusuf Isyaku, Babatunde Samuel Obadawo, Hadiza Abdulrahman Lawal, Samuel Akolade Ajibowu

**Affiliations:** Department of Chemistry, Faculty of Physical Sciences, Ahmadu Bello University, Zaria, Nigeria

**Keywords:** Colorectal cancer, Cyclin dependent kinase 2 enzyme, Computer-aided drug design, Quantitative structure activity relationship, Molecular docking, Imidazole

## Abstract

**Background:**

Colorectal cancer is common to both sexes; third in terms of morbidity and second in terms of mortality, accounting for 10% and 9.2% of cancer cases in men and women globally. Although drugs such as bevacizumab, Camptosar, and cetuximab are being used to manage colorectal cancer, the efficacy of the drugs has been reported to vary from patient to patient. These drugs have also been reported to have varying degrees of side effects; thus, the need for novel drug therapies with better efficacy and lesser side effects. In silico drugs design methods provide a faster and cost-effect method for lead identification and optimization. The aim of this study, therefore, was to design novel imidazol-5-ones via in silico design methods.

**Results:**

A QSAR model was built using the genetic function algorithm method to model the cytotoxicity of the compounds against the HCT116 colorectal cancer cell line. The built model had statistical parameters; *R*^2^ = 0.7397, *R*^2^_adj_ = 0.6712, *Q*^2^_cv_ = 0.5547, and *R*^2^_ext._ = 0.7202 and revealed the cytotoxic activity of the compounds to be dependent on the molecular descriptors nS, GATS5s, VR1_Dze, ETA_dBetaP, and L3i. These molecular descriptors were poorly correlated (VIF < 4.0) and made unique contributions to the built model. The model was used to design a novel set of derivatives via the ligand-based drug design approach. Compounds e, h, j, and l showed significantly better cytotoxicity (IC_50_ < 5.0 μM) compared to the template. The interaction of the compounds with the CDK2 enzyme (PDB ID: 6GUE) was investigated via molecular docking study. The compounds were potent inhibitors of the enzyme having binding affinity of range −10.8 to −11.0 kcal/mol and primarily formed hydrogen bond interaction with lysine, aspartic acid, leucine, and histidine amino acid residues of the enzyme.

**Conclusion:**

The QSAR model built was stable, robust, and had a good predicting ability. Thus, predictions made by the model were reliably employed in further in silico studies. The compounds designed were more active than the template and showed better inhibition of the CDK2 enzyme compared to the standard drugs sorafenib and kenpaullone.

## Background

Cancer is a major public health problem worldwide and is the second leading cause of death in the USA. Cancer is a term that refers to over 200 independent health conditions in which cells in different body parts divide abnormally and uncontrollably. Four of the most common cancers are lung, breast, prostate and colorectal cancer [1]. Colorectal cancer (CRC) is the third most prevalent cancer in both sexes and the second in terms of the rate of mortality. The cancer accounts for 10% and 9.2% of all cancer cases in men and women globally and causes over 500,000 deaths annually. In the USA, about 147,950 new cases and an estimated 53,200 mortalities from CRC are expected in the year 2020. While in Germany, 1 in 14 men and 1 in 18 women would diagnosed with CRC within their lifetime, and 1 in 32 men and 1 in 39 women will die from CRC [1, 2].

CRC results from the abnormal division and growth of colon cells. This abnormal division of cells forms polyps which may be benign or cancerous. The cause of these abnormal divisions is yet to be fully understood [3]. However, risk factors have been associated with age, race, family history, and a sedentary lifestyle. CRC is more frequently diagnosed in persons over the age of 50, persons of African descent, or persons who consume high amounts of tobacco, alcohol, and high-fat diets. Persons with prior health conditions such as obesity and diabetes, are also more susceptible to the cancer [3–5]. However, over a fourth of CRC cases are attributed to hereditary factors. The most common forms of hereditary colorectal cancer are familial adenomatous polyposis coli (FAP) and hereditary non-polyposis colon cancer (HNPCC, Lynch syndrome) [2]. Just like most cancers, CRC at its infancy shows no significant symptoms, thus making early detection difficult. However, where symptoms exist, they include changes in bowel frequency, rectal bleeding, abdominal pains, weakness, and weight loss [6]. Diagnosis of CRC can be made by sigmoidoscopy or by colonoscopy while treatment methods include surgery, radiation therapy and drug treatments, such as chemotherapy, targeted therapy, and immunotherapy [2, 7].

Drug treatment of CRC employs various strategies, one such strategy is the use of cytotoxic agents, such as 5-fluorouracil, oxaliplatin, and irinotecan. Another drug treatment strategy involves the use of compounds that block certain CRC targets. CRC chiefly targets the epidermal growth factor receptor (EGFR) and the vascular endothelial growth factor (VEGF). Thus, compounds such as cetuximab, bevacizumab, and ramucirumab, which block these targets are commonly employed in the fight against CRC [2, 8]. The human cyclin-dependent kinase 2 (CDK2) has also been observed to be overexpressed in CRC patients, thus, inhibition and/or downregulation of this kinase has also emerged as a strategy for tackling CRC [9, 10].

CDK2 primarily binds to cyclins A, B, and E and plays an important role in cell cycle regulation. It is responsible for G1 to S phase transition in the cell cycle. In normal healthy cells, CDK2 is dispensable as CDK1 plays mimicking roles. In cancerous cells, however, CDK2 plays a pivotal role in cell growth and progression [9, 11]. Overexpression of CDK2 and cyclins A and E has been observed in ovarian, colorectal, breast, prostate, and lung cancer patients [9, 10]. Therefore, drugs such as flavopiridol, roscovitine, olomoucine, adapalene, and kenpaullone, which are reported to be CDK2 inhibitors and sorafinib, aspirin (salicylic acid), etc., which have been reported to cause downregulation of the enzyme via various mechanisms have been employed as therapies for these cancers [9, 12–14]. However, the non-specificity and toxicity of most of these drugs ensure that the search for more specific and less toxic candidates goes on [15]. Thus, this study built a robust quantitative structure activity relationship (QSAR) model which predicted the cytotoxic activity of imidazol-5-one compounds against HCT-116 CRC cell line, then, the built model was used to design a novel set of imidazole-5-ones compounds. Furthermore, the interaction between the designed compounds and the CDK2 enzyme was carried out via the molecular docking approach to determine the potential of the compounds to be used as inhibitors of the enzyme.

## Methods

### QSAR

#### Dataset

**Fig. 1:**
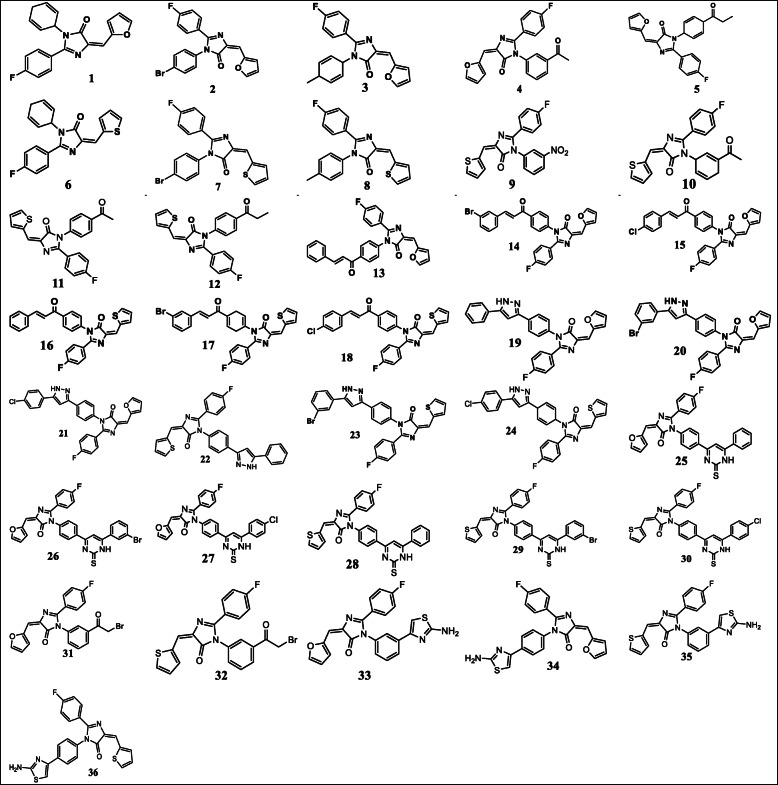
The structures of the compounds

A series of thirty-six (36) imidazole-5-ones compounds reported to have cytotoxic activity against HCT-116 colorectal cancer cell line were obtained from the literature [16]. The structures of the compounds were obtained, drawn, and are presented in Fig. 1. The activity of the compounds (IC_50_) ranged from 4.87-97.18 μM. The skew in the activity of the compounds was minimized by converting the activity to their logarithmic scale equivalents using the formula (pIC_50_ = −log_10_ IC_50_) [17]. In the logarithmic scale, the cytotoxic activity (pIC_50_) of the compounds ranged from 4.02-5.31 M.

#### Geometry optimization, calculation of molecular descriptors, and data division

The 2D structure of the obtained compounds was drawn using the ChemDraw Professional v. 16.0 software. The 2D structures were converted to their 3D forms on the Spartan 14 V 1.1.4 software. The ground state equilibrium structures of the compounds were obtained via geometry optimization using the density functional theory’s B3LYP/6-31G* basis set [18, 19]. The molecular descriptors of the designed compounds were calculated using the PaDEL-Descriptor v 2.21 software. One thousand eight hundred and seventy-five 1D, 2D, and 3D molecular descriptors were calculated for each compound. These molecular descriptors were further pretreated at a correlation cut-off of 0.7 using the DTC Lab Data Pretreatment v 1.2 software. Data pretreatment was carried out to remove highly correlated and redundant molecular descriptors [20]. The pretreated molecular descriptors were subsequently divided into a training and test set using the Kennard-Stone algorithm integrated into the DTC Lab Data Division v 1.2 software. The training set consists of 25 (70%) randomly selected compounds while the test set is made up of the remaining 11 (30%) of the compounds in the dataset [18, 21]. The training set was used to build the model while the test set was used in the external validation of the built model.

#### Model building and validation

The dataset of molecular descriptors of the training set compounds was transferred to the Biovia Materials Studio v 8.0 software. The QSAR model was subsequently built using the Genetic Function Algorithm function in the abovementioned Materials Studio software [18].

The stability, reproducibility, and predicting ability of the model were ascertained by subjecting the model to appropriate validation tests. The stability of the model was determined by carrying out a multilinear regression and one-way ANOVA on the molecular descriptors present in the model using the Microsoft Excel 2016 software. Furthermore, the inter-correlation between the molecular descriptors in the model was determined by calculating the variance inflation factor (VIF). VIF was obtained as the leading diagonal of the inverse of the correlation matrix [20]. The external validation of the model was carried out using Equation 1:

1 $$ {R^2}_{ext}=\frac{\sum_{i=1}^{11}{\left({Y}_{{\mathit{\exp}}_i}-{Y}_{pred_i}\right)}^2}{\sum_{i=1}^{11}{\left({Y}_{{\mathit{\exp}}_i}-{\overline{Y}}_{pred_{train}}\right)}^2} $$

Where *R*^2^_*ext*_ is the external coefficient of determination, *Y*_*expi*_ and *Y*_*predi*_ are the experimental and predicted cytotoxic activity of the *i*th test set compound while ${{\overline{Y}}_{exp}}_{train}$ is the mean of the experimental cytotoxic activity of the training set compounds [17]. The robustness of the built model was evaluated by subjecting the training set to a *Y* randomization test. In the test, a series of randomly generated multilinear regression (MLR) models are generated by shuffling the molecular descriptors which keeping the cytotoxic activity constant [18]. For a robust model, the adopted validation parameters of *R*^2^ and *Q*^2^ are larger than those randomly generated by the *Y* randomization test. Furthermore, the *Y* randomization coefficient of determination (${cP}_p^2$) for a robust model is greater than 0.5 [18]. The *Y* randomization test was carried out using the DTC Lab’s YRandomization software version 1.2. The zone of applicability of the model was graphed out using the leverages approach as described by Ikwu et al. [22].

### Ligand-based design

Ligand-based design was employed in designing novel imidazol-5-one derivatives. A template (compound 20) was selected upon which further modifications were made. Compound 20 was selected as a template because it had high cytotoxic activity, a low residual and was well within the applicability domain of the built model [27]. The designed compounds were drawn, optimized, and their molecular descriptors were calculated as described in the “Geometry optimization, calculation of molecular descriptors and data division” section. The relevant molecular descriptors were then copied and inserted into the regression equation generated for the model to predict the cytotoxic activity of the designed compounds [23].

### Molecular docking

#### Preparation of enzyme and ligands

**Plate 1:**
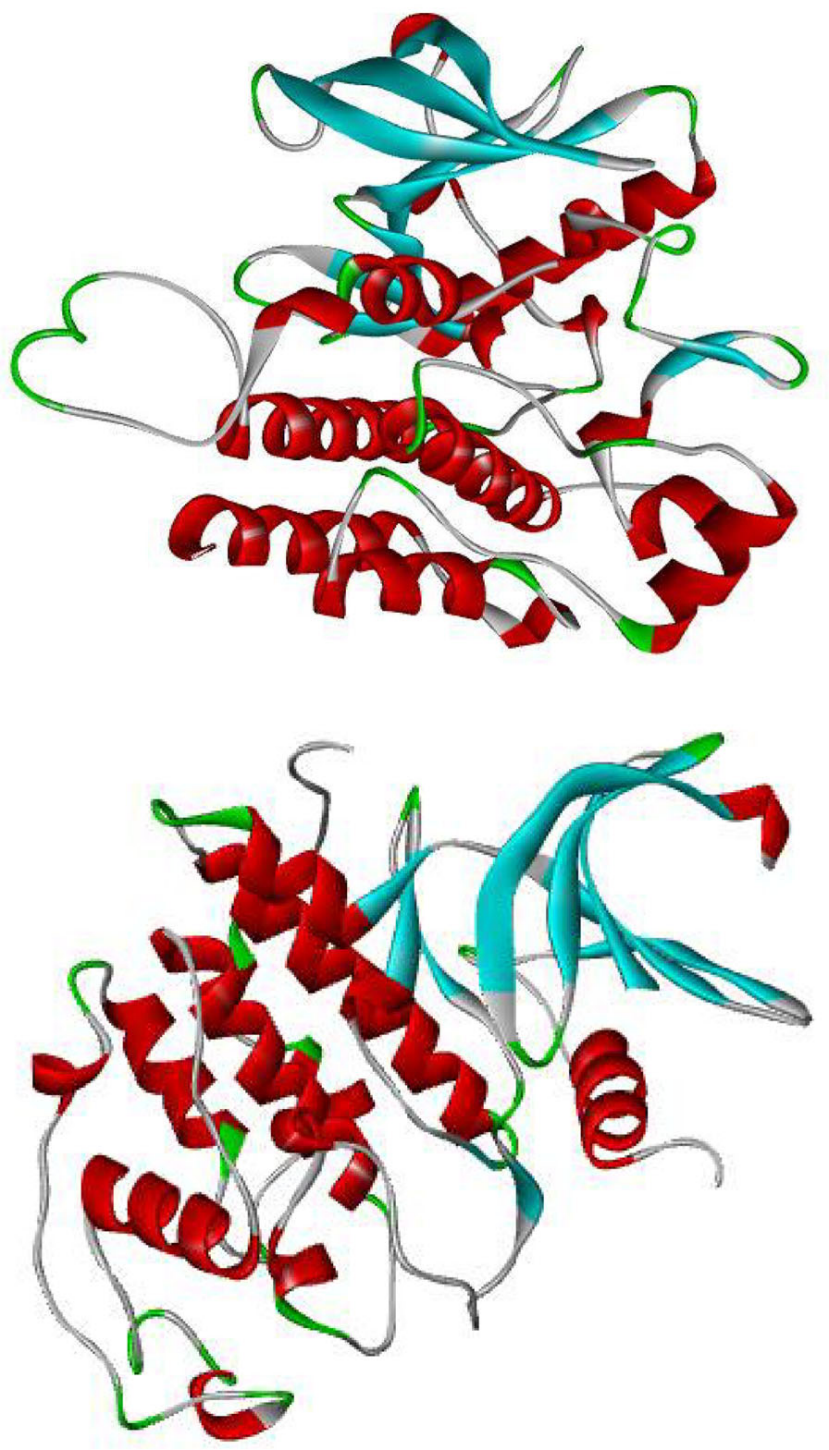
Crystal structure of CDK2 enzyme (PDB ID: 6GUE)

The crystal structure of the human cyclin-dependent kinase 2; CDK2 (PDB ID: 6GUE) was obtained from the protein data bank. The downloaded enzyme was prepared using the Discovery Studio 2016 software; the water molecules and ligands present in the downloaded enzyme were deleted. All chains excluding the A chain of the downloaded enzyme were also removed while preparing the enzyme for molecular docking studies [30]. The crystal structure of the prepared enzyme is presented in Plate 1.

The ligands were prepared for molecular docking by converting the equilibrium structures to their protein data bank (.pdb) file formats using the Spartan 14 v 1.1.4 software [30]. Sorafenib has been reported to downregulate CDK2 [14] while kenpaullone has been reported to inhibit CDK2 [12]. Thus, the structures of sorafenib (PubChem CID: 216239) and kenpaullone (PubChem CID: 3820) were obtained, drawn, optimized, and saved in the .pdb file format to prepare them for molecular docking study.

#### Enzyme—ligand interaction

The prepared enzyme and ligands were transferred to the PyRx software and their binding interaction was monitored. The binding affinity and the interaction files (.pdbqt file format) were obtained [24]. The interaction files obtained were subsequently opened in the Discovery Studio software where the visualization of the interaction was carried out [18].

## Results

### QSAR

A QSAR model was built to predict the cytotoxic activity of imidazole-5-one compounds against HCT-116 colorectal cancer cell line. The regression equation of the built model is presented in Equation 2. The regression statistics of the model is presented in Table 1 while the model validation parameters are presented in Table 2. The external validation of the built model was calculated in Supplementary Table S1. The definition of the molecular descriptors in the build regression model is presented in Table 3 while Table 4 presents the statistical parameters of these molecular descriptors. The result of the *Y* randomization test is presented in Table 5.

**Table 1:** Regression statistics

	df	SS	MS	*F*	*p value*
Regression	5	2.349644479	0.469928896	10.79888	4.96E-05
Residual	19	0.8268128	0.043516463		
Total	24	3.176457278			

Key: *df* degree of freedom, *SS* sum of squares, *MS* mean square error; *F* F statistic

**Table 2:** Model validation parameters

Parameter	Model 1	Benchmark^a^
Friedman LOF	0.2006	
R-squared (*R*^2^)	0.7397	≥ 0.6
Adjusted R-squared (*R*^2^_adj._)	0.6712	≥ 0.6
Cross validated R-squared (*Q*^2^_cv_)	0.5547	≥ 0.5
Significant regression?	Yes	Yes
External validation (*R*^2^_ext._)	0.7202	≥ 0.6

Key: ^a^[31]

**Table 3:** Name, definition, category and class of molecular descriptors

Name	Definition	Category	Class
nS	Number of sulfur atoms	Atom count descriptor	2D
GATS5s	Geary autocorrelation—lag 5/weighted by *I* state	Autocorrelation descriptor	2D
VR1_Dze	Randic-like eigenvector-based index from Barysz matrix/weighted by Sanderson electronegativities	Barysz matrix descriptor	2D
ETA_dBetaP	A measure of relative unsaturation content relative to molecular size	Extended topochemical atom descriptor	2D
L3i	3rd component size directional WHIM index/weighted by relative first ionization potential	PaDEL WHIM descriptor	3D

**Table 4:** Statistical parameters of descriptor

Descriptor	Coefficient	ME	VIF	*t* stat	*p* value	SE
nS	−0.1249	0.053941	1.174334	−1.49834	0.150477	0.0833
GATS5s	−5.1376	2.446025	3.119743	−4.64905	0.000175	1.1051
VR1_Dze	0.0003	−0.0787	1.12511	3.097051	0.005934	0.0001
ETA_dBetaP	12.7903	−1.50015	2.717113	5.457748	2.89E-05	2.3435
L3i	−0.1736	0.078893	1.517541	−1.72007	0.101668	0.1009

Key: *ME* mean effect, *VIF* variance inflation factor, *SE* standard error

**Table 5:** *Y* randomization test

Model	*R*	*R*^2^	*Q*^2^
Original	0.860062	0.739706	0.554712
Random 1	0.569068	0.323839	−1.17969
Random 2	0.556022	0.309161	−0.34937
Random 3	0.427541	0.182791	−0.53108
Random 4	0.501596	0.251598	−0.85524
Random 5	0.645348	0.416475	0.063014
Random 6	0.502871	0.252879	−0.52581
Random 7	0.522143	0.272633	−0.24368
Random 8	0.595235	0.354305	−1.92796
Random 9	0.423402	0.179269	−0.35291
Random 10	0.588755	0.346633	−0.06237
Random models parameters			
Average *R*	0.533198		
Average *R*^2^	0.288958		
Average *Q*^2^	−0.59651		
cRp^2^	0.580402		

**Fig. 2:**
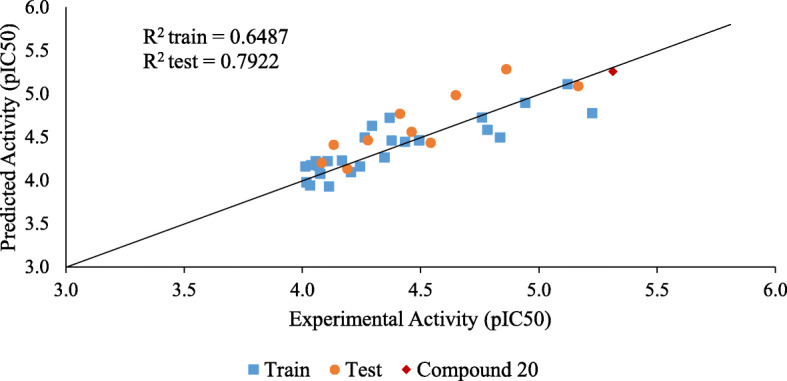
Experimental and predicted cytotoxic activity

**Fig. 3:**
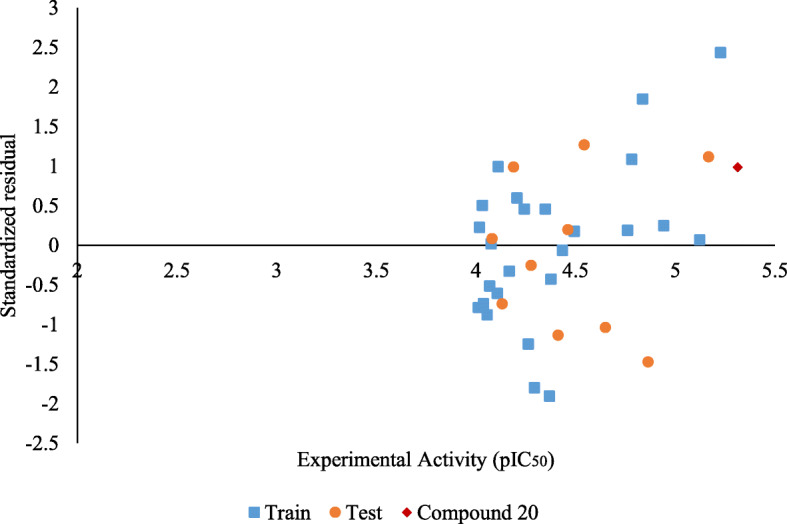
Standardized residual against experimental activity

**Fig. 4:**
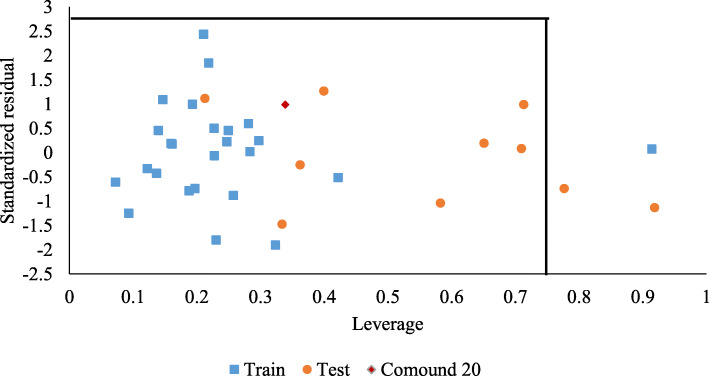
The zone of applicability of QSAR model

The predicting ability of the built model was ascertained by comparing the experimental and predicted cytotoxic activity of the compounds in the dataset. The experimental and predicted cytotoxicity of the compounds in the training and test are presented in Supplementary Table S2 and S3, respectively. Figure 2, however, presents a scatter plot of these experimental and predicted cytotoxic activities. While Fig. 3 presents a scatter plot of the experimental activity against the standardized residual for each molecule in the dataset, the zone of applicability of the model was graphed out using the leverages method [18] and plotted in Fig. 4.

2 $$ {\displaystyle \begin{array}{l}{pIC}_{50}=-0.124851343\times nS-5.137570104\times GATS5s+0.000256939\times VR1\_ Dze+\\ {}12.790319005\times ETA\_ dBetaP-0.173566533\times L3i+6.319854761\end{array}} $$

### Ligand-based design

Compound 20 was selected as the template used in ligand-based design. Slight modifications were made to it and twelve derivatives were designed. The molecular descriptors of the designed compounds were obtained and inserted into Equation 2 to obtain their cytotoxicity. These molecular descriptors are presented in Supplementary Table S4. The designed compounds and their corresponding activities are presented in Table 6.

**Table 6:** Cytotoxic activity of designed compounds

Compound	*R*_1_	*R*_2_	*R*_3_	pIC_50_ (M)	IC_50_ (μM)
a.	-H	-Br	-H	5.1581	6.9490
b.	-H	-NO_2_	-H	5.2999	5.0131
c.	-F	-H	-H	5.3681	4.2843
d.	-Cl	-H	-H	5.1022	7.9040
e.	-F	-H	-F	5.4049	3.9362
f.	-Cl	-H	-Cl	4.9666	10.7985
g.	-Br	-Br	-H	5.1224	7.5440
h.	-F	-F	-H	5.8668	1.3590
i.	-Cl	-Cl	-H	4.9438	11.3808
j.	-NO_2_	-H	-H	5.478	3.3265
k.	-Br	-H	-Br	5.1535	7.0224
l.	-NO_2_	-H	-NO_2_	5.7351	1.8403
Template (compound 20)	-Br	-H	-H	5.257	5.5330
Doxorubicin^a^					5.23 ± 0.2

Key: ^a^ Abo-Elanwar et al. [16]

### Molecular docking study

**Plate 2:**
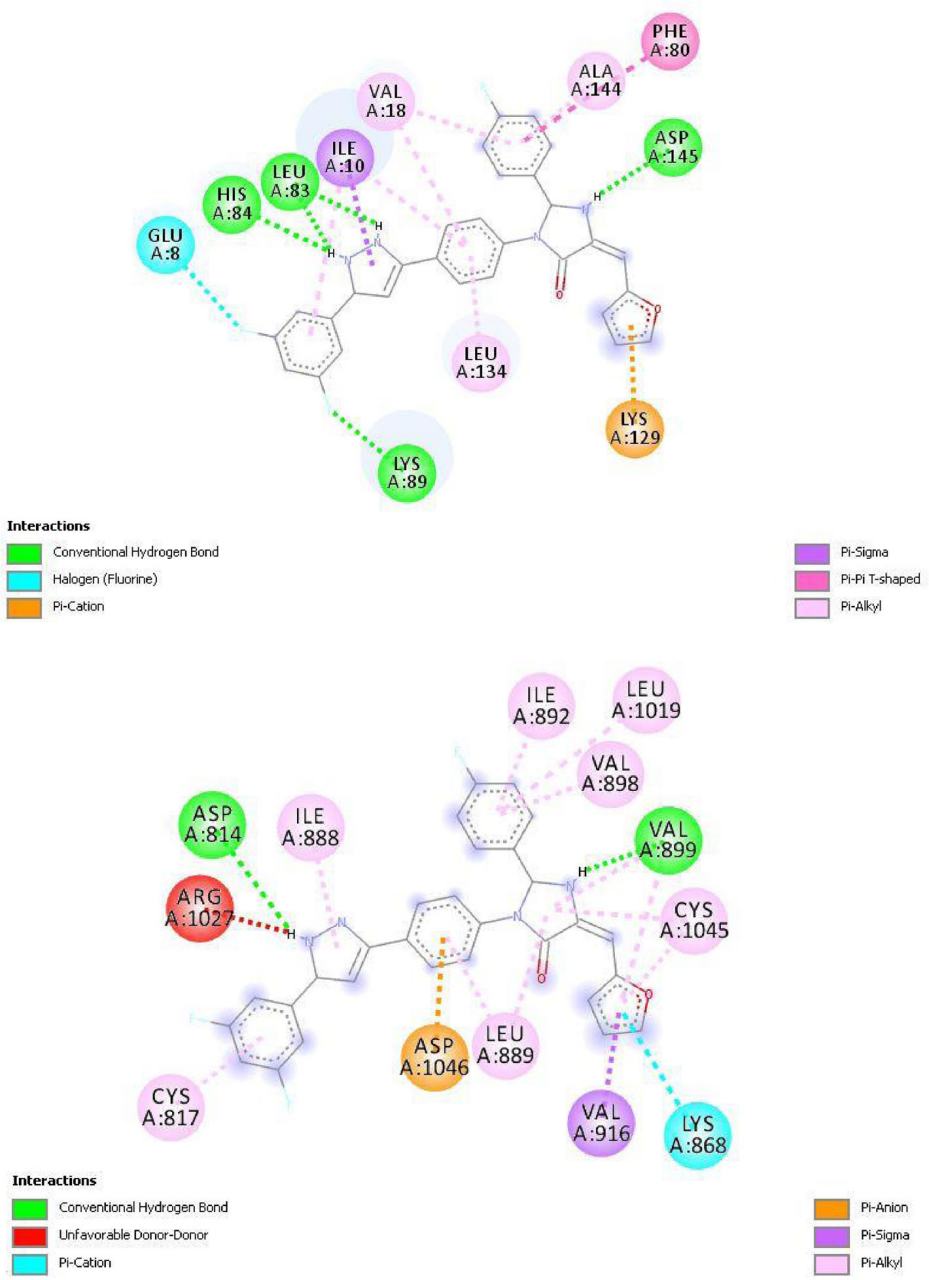
2D interaction between compound e and CDK2 enzyme (PDB code: 6GUE)

**Plate 3:**
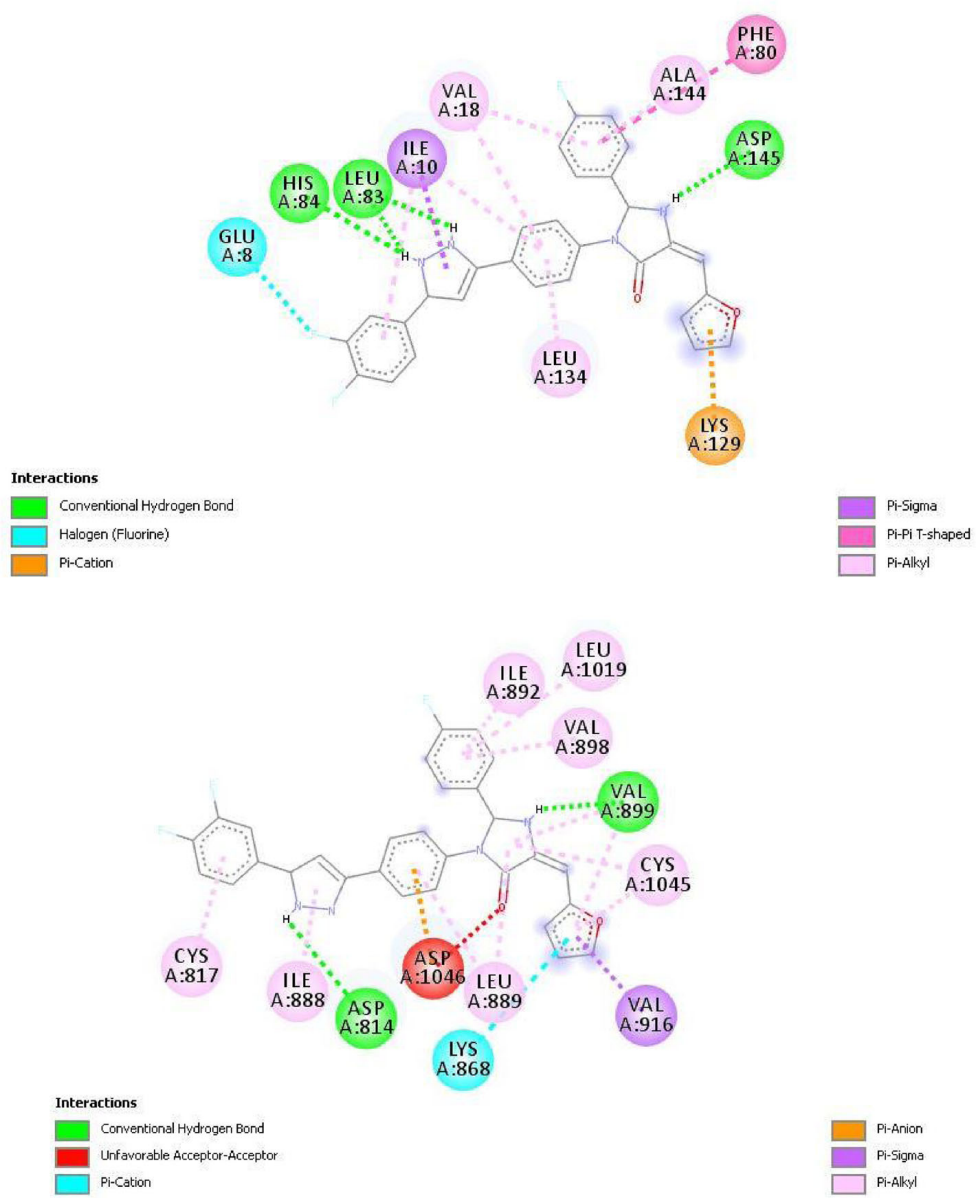
2D interaction between compound h and CDK2 enzyme (PDB code: 6GUE)

The crystal structure of the human cyclin-dependent kinase 2 (PDB ID: 6GUE) was downloaded from the protein databank. The downloaded kinase was prepared using the Discovery Studio 2016 software. The A chain of the downloaded receptor was employed in molecular docking studies using the PyRx software. Multiple enzyme-ligand interactions were carried out by the software and the most stable conformation was presented by the software. Compounds e, h, j, and l were observed to have significantly better cytotoxic activity compared to the template compound and standard drug doxorubicin. Thus, the molecular docking study of these compounds and the template were carried out with the CDK2 enzyme. The standard drugs sorafenib and kenpaullone were also docked with the CDK2 enzyme and their binding affinity was compared with those of the designed compounds. Findings from the molecular docking study are presented in Table 7. Compounds e and h had the highest binding affinity with a value of −11.0 kcal/mol each. The 2D interaction of compounds e and h and the enzyme are presented in Plates 2 and 3, respectively.

**Table 7:** Docking interaction between ligand and enzyme

Molecule	Binding Affinity (kcal/mol)	Interactions			
		Hydrogen bond	Electrostatic	Hydrophobic	Others
e	−11.0	LYS89, ASP145, LEU83, HIS84	LYS129	ILE10, PHE80, VAL18, LEU134, ALA144	Halogen (fluorine) interaction (GLU8)
h	−11.0	ASP145, LEU83, HIS84	LYS129	ILE10, PHE80, VAL18, LEU134, ALA144	Halogen (fluorine) interaction (GLU8)
j	−10.8	LYS89, ASP145, LEU83, HIS84	LYS129	ILE10, PHE80, GLY11, VAL18, LEU134, ALA144	
l	−10.8	LYS20, LYS89, ASP145, LEU83, HIS84	LYS129	ILE10, PHE80, VAL18, LEU134, ALA144	
20	−10.6	LEU83, ASN132 ASP145 LYS129	LYS129	ILE10, TYR15, LEU134, VAL18, ALA31,	
Sorafenib	−9.7	TYR15, ASN132 ASP145, LEU83, GLY13, HIS84	LYS129	TYR15, ALA144, ASP145, VAL18,	Halogen (fluorine) interaction (GLY13)
Kenpaullone	−9.4	LEU83		LEU134, VAL18, ALA144, ILE10

## Discussion

### QSAR

QSAR is a variant of the quantitative structure-property relationship approach. This variant posits that the pharmacological activity of a dataset of compounds can be expressed as a linear combination of their molecular descriptors [25]. The built model revealed that the cytotoxic activity of imidazole-5-ones against HCT116 colorectal cancer cell line was strongly dependent on the molecular descriptors nS, GATS5s, VR1_Dze, ETA_dBetaP, and L3i. The regression equation of the model was shown in Equation 2 while the definition of the molecular descriptors was presented in Table 3. The model built was observed to be significant, possessing *p* < 0.05 (Table 1). The model also met all internal and external validation benchmarks (Table 2). The predictions made by the model are therefore reliable, and can be employed in further theoretical and experimental studies.

A robust model is characterized by the presence of poorly correlated molecular descriptors. This ensures that each molecular descriptor makes a unique contribution to the prediction made by the model [17]. Poorly correlated molecular descriptors have VIF values < 5.0. As shown in Table 4, the molecular descriptors were all poorly correlated (VIF < 3.5). This implies that each molecular descriptor made a unique contribution to the model. However, the contribution of the descriptors nS and L3i were observed to the insignificant (*p* value > 0.05; Table 4). This was also mirrored by their low mean effects (< 0.02; Table 4). The molecular descriptors GATS5s and ETA_dBetaP (*p* < 0.05) were the major contributors to the model, both had |ME| > 1.0.

The predicting ability of the model was determined by using the regression equation to predict the cytotoxic activity of the compounds in the training and test set. The predicted activity and residual of each compound in the train and test were presented in Supplementary Tables S1 and S2. Figure 2 however presented the scatter plot of the experimental and predicted cytotoxic activity of the compounds in the dataset. The coefficient of determination for both the test and train set were both > 6.0. Thus implying that the model had a good predicting ability. The presence of systematic errors in the model was investigated using the standardized residual method. Figure 3 presented a graph of the standardized residual against experimental activity. The data points were observed to be randomly distributed, thus, signifying the absence of systematic errors [18]. The robustness of the model was determined using the *Y* randomization test. Findings were presented in Table 5. The *Y* randomization co-efficient of determination (${cP}_p^2$) was observed to be greater than 0.5, thus implying that the model built was a robust one [17]. The zone of applicability of the model was graphed out using the leverages method and was presented in Fig. 4. The zone of applicability presents those molecules whose cytotoxic activity was satisfactorily predicted by the model. Compounds within the zone of applicability can be satisfactorily employed for further in silico studies [22, 26].

### Ligand-based design

Ligand-based design is one of two methods used in computer-based drug design. In this design method, a compound of known pharmacological activity is obtained, and slight modifications are made to this compound to obtain a novel set of compounds with potentially better pharmacological activity [27]. In this study, compound 20 was selected as the template compound, because it was very active (IC_50_ = 4.87 μM), the model made a good prediction of its activity (Fig. 2) and it was also well within the zone of applicability of the model (Fig. 4). It was observed that the cytotoxicity of the compounds was dependent on the electronegativity of the substituent groups [16]. Thus, modification of compound 20 primarily involved the introduction of electronegative substituents.

The activity of the compounds was observed to increase significantly when fluorine (-F) and Nitro (-NO_2_) groups are used as substituent. The activity of the compounds also increased with increase in the number of these substituents. Compounds e and h (two -F substituents) for instance were more active than compound c (one -F substituent). Similarly, compound l (two -NO_2_ substituents) was more active compared to compound b (one -NO_2_ substituent). Six compounds (b, c, e, j, l, and h) were more active than the template compound and the standard drug doxorubicin (Table 6).

### Molecular docking study

Molecular docking investigates the interaction between a compound (ligand) and its target (enzyme) at an atomic level. It investigates the binding affinity, nature, and type of bonding and nonbonding interactions between the ligand and amino acid residues of the enzyme [28, 29]. Table 7 presented the results of docking studies between compounds e, h, j, and l, and the CDK2 enzyme. The template compound (compound 20) and the standard drugs sorafenib and kenpaullone were also docked with the CDK2 enzyme. The binding affinity of the designed compounds (−10.8 to −11.0 kcal/mol) was slightly higher than that of the template (−10.6 kcal/mol) and was significantly higher than that of sorafenib (−9.7 kcal/mol) and kenpaullone (−9.4 kcal/mol). The designed compounds formed hydrogen bonds with lysine (LYS20, LYS89), aspartic acid (ASP145), leucine (LEU83), and histidine (HIS84) amino acid fraction of the CDK2 enzyme. The hydrogen interaction formed was primarily with the pyrrolidine and 2, 3-dihydro-1H-pyrrole groups of the designed compounds. Compounds e and h had higher binding affinities (−11.0 kcal/mol each) and this could be attributed to the halogen (fluorine) interaction formed between these compounds and the glutamic acid (GLU8) residue of the CDK2 enzyme. All compounds however formed an electrostatic interaction with the lysine (LYS129) amino acid residue of the enzyme.

## Conclusion

The cytotoxic activity of imidazol-5-ones against HCT116 colorectal cancer cell line was modeled via the QSAR approach. The cytotoxic activity of the compounds was dependent on the molecular descriptors nS, GATS5s, VR1_Dze, ETA_dBetaP, and L3i. Novel set of compounds were designed via the ligand-based design approach, and their activity was strongly dependent on the electronegativity of the substituent group(s). Compounds e, j, h, and l showed remarkable cytotoxicity (< 4.0 μM) against the colorectal cancer cell line. The compounds were also observed to be potent inhibitors of the CDK2 enzyme, having binding affinities ranging from −10.8 to −11.0 kcal/mol and forming hydrogen bond interactions with lysine, aspartic acid, leucine, and histidine amino acid residues. The designed compounds were predicted to be more potent than the standard drugs doxorubicin and had a higher binding energy compared to the standard drugs sorafenib and kenpaullone.

## Supplementary information

**Additional file 1: Table S1**. External validation of built model. **Table S2**. Experimental, predicted and residual cytotoxic activity of training set compounds. **Table S3**. Experimental, predicted and residual cytotoxic activity of test set compounds. **Table S4**. Molecular descriptors and predicted cytotoxic activity of designed compounds

## Data Availability

The dataset of molecular descriptors generated and analyzed during the current study are included in this published article and its supplementary information files.

## Abbreviations

CRC: Colorectal cancer
CDK2: Cyclin dependent kinase 2
VEGF: Vascular endothelial growth factor receptor
EGFR: Epidermal growth factor receptor
QSAR: Quantitative structure activity relationship
B3LYP: Becke, 3-parameter

## References

[1] Siegel RL, Miller KD, Jemal A (2020). Cancer statistics, 2020.

[2] Kolligs FT (2016). Diagnostics and epidemiology of colorectal cancer. Visceral Medicine.

[3] Simon L, Imane A, Srinivasan KK, Pathak L, Daoud I (2016) In silico drug-designing studies on flavanoids as anticolon cancer agents: pharmacophore mapping, molecular docking, and Monte Carlo method-based QSAR modeling. Interdiscip Sci Comput Life Sci. 10.1007/s12539-016-0169-4 10.1007/s12539-016-0169-4 27059855

[4] Araghi M, Soerjomataram I, Jenkins M, Brierley J, Morris E, Bray F, Arnold M (2019). Global trends in colorectal cancer mortality: projections to the year 2035. Int J Cancer.

[5] Qawoogha, S. S. and Shashiwala, A. (2020). Identification of potential anticancer phytochemicals against colorectal cancer by structure-based docking studies. Journal of Receptors and Signal Transduction¸ 10.1080/10799893.2020.1715431 10.1080/10799893.2020.1715431 31971455

[6] Bekkink MO, McCowan C, Falk GA, Teljeur C, Van de Laar FA, Fahey T (2010). Diagnostic accuracy systematic review of rectal bleeding in combination with other symptoms, signs and tests in relation to colorectal cancer. Br J Cancer.

[7] Mármol I, Sánchez-de-Diego C, Dieste A, Cerrada E, Yoldi R (2017). Colorectal carcinoma: a general overview and future perspectives in colorectal cancer. Int J Mol Sci.

[8] Clarke JM, Hurwitz HI (2013). Targeted inhibition of VEGF receptor 2: an update on ramucirumab. Expert Opin Biol Ther.

[9] Shi X, Li H, Yao H, Liu X, Li L, Leung K, Kung H, Lin M (2015). Adapalene inhibits the activity of cyclin-dependent kinase 2 in colorectal carcinoma. Mol Med Rep.

[10] Tadesse S, Anshabo AT, Portman N, Lim E, Tilley W, Caldon E, Wang S (2019) Targeting CDK2 in cancer: challenges and opportunities for therapy. Drug Discov Today 10.1016/j.drudis.2019.12.001 10.1016/j.drudis.2019.12.001 31839441

[11] Wood DJ, Korolchuk S, Tatum NJ, Wang LZ, Endicott JA, Noble ME, Martin MP (2018). Differences in the conformational energy landscape of CDK1 and CDK2 suggest a mechanism for achieving selective CDK inhibition. Cell Chemical Biology.

[12] Cicenas J, Kalyan K, Sorokinas A, Stankunas E, Levy J et al (2015) Roscovitine in cancer and other diseases. Annals of Translational Medicine 3(10) 10.3978/j.issn.2305-5839.2015.03.61 PMC4486920 26207228

[13] Dachineni R, Ai G, Kumar R, Sadhu S, Tummala H, Bhat J (2015). Cyclin A2 and CDK2 as novel targets of aspirin and salicylic acid: a potential role in cancer prevention. Mol Cancer Res.

[14] Oh, S. J., Erb, H. H., Hobisch, A., Santer, F. R. and Culig, Z. (2012). Endocrine-related cancer, 19, 305 – 319. 10.1530/ERC-11-0298 10.1530/ERC-11-0298 PMC3353237 22383427

[15] Tadesse S, Caldon E, Tilley W, Wang S (2018) Cyclin dependent kinase 2 inhibitors in cancer therapy: an update. J Med Chem 10.1021/acs.jmedchem.8b01469 10.1021/acs.jmedchem.8b01469 30543440

[16] Abo-Elanwar Y, Mostafa AS, El-Sayed MA, Nasr MN (2019). Synthesis and biological evaluation of new 2-(4-fluorophenyl) imidazol-5-ones as anticancer agents. Journal of Applied Pharmaceutical Science.

[17] Adeniji, S. E., Uba, S., Uzairu, A. and Arthur, D. E. (2019). A derived QSAR model for predicting some compounds as potent antagonist against Mycobacterium tuberculosis: a theoretical approach. Hindawi, 10.1155/2019/5173786 10.1155/2019/5173786 PMC6521565 31186969

[18] Abdullahi M, Uzairu A, Shallangwa GA, Mamza P, Arthur DE, Ibrahim MT (2019) An Insilico modelling study on some C14-urea-tetrandrine derivatives as potent anti-cancer against prostate (PC3) cell line. Journal of King Saud University – Science 10.1016/j.jksus.2019.01.008

[19] Becke AD (1993). Becke’s three parameter hybrid method using the LYP correlation functional. J Chem Phys.

[20] Ibrahim, M. T., Uzairu, A., Shallangwa, G. A. and Ibrahim, A. (2018). In-silico studies of some oxadiazoles derivatives as anti-diabetic compounds. Journal of King Saud University –Science. 10.1016/j.jksus.2018.06.006.

[21] Kennard RW, Stone LA (1969). Computer aided design of experiments. Technometrics.

[22] Ikwu FA, Shallangwa GA, Mamaza AP, Uzairu A (2020). In silico studies of piperazine derivatives as potent anti-proliferative agents against PC-3 prostate cancer cell lines. Heliyon.

[23] Wilson GL, Lill M (2011) Integrating structure-based and ligand-based approaches for computational drug design. Future Med Chem 3(6) 10.4155/fmc.11.18 10.4155/fmc.11.18 21554079

[24] Trott O, Olson AJ (2010) AutoDock Vina: improving the speed and accuracy of docking with a new scoring function, efficient optimization and multithreading. J Comput Chem 31 10.1002/jcc.21334 PMC3041641 19499576

[25] Golbraikh A, Wang XS, Zhu H, Tropsha A, Leszczynski J (2016). Predictive QSAR modeling: methods and applications in drug discovery and chemical risk assessment. Handbook of Computational Chemistry.

[26] Netzeva TI, Worth A, Aldenberg T (2005). Current status of methods for defining the applicability domain of (quantitative) structure-activity relationships. ATLA.

[27] Aparoy P, Reddy KK, Reddana P (2012). Structure and ligand based drug design strategies in the development of novel 5-LOX inhibitors. Curr Med Chem.

[28] Guedes I, Magalhäes C, Dardenner L (2014). Receptor – ligand molecular docking. Biophys Rev.

[29] Meng X, Zhang H, Mezei M, Cui M (2011). Molecular docking: a powerful approach for structure-based drug discovery. Curr Comput Aided Drug Des.

[30] Arthur DE, Uzairu A, Mamza P, Abechi SE, Shallangwa GA (2018) In silico modelling of quantitative structure-activity relationship of Pgi50 anticancer compounds on k-562 cell line. Cogent Chem 4(1) 10.1080/23312009.2018.1432520

[31] Tropsha A (2010). Best practices for QSAR model development, validation and exploitation. Molecular Informatics.

