# Supplementary material for: In silico design and molecular docking study of CDK2 inhibitors with potent cytotoxic activity against HCT116 colorectal cancer cell line
Source: J Genet Eng Biotechnol. 2020 Sep 15;18:51. doi: 10.1186/s43141-020-00066-2 (PMC7492310; doi:10.1186/s43141-020-00066-2)
Supplement: Supplementary file 1 — Additional file 1: Table S1. External validation of built model. Table S2. Experimental, predicted and residual cytotoxic activity of training set compounds. Table S3. Experimental, predicted and residual cytotoxic activity of test set compounds. Table S4. Molecular descriptors and predicted cytotoxic activity of designed compounds [file 43141_2020_66_MOESM1_ESM.docx]

**IN SILICO DESIGN AND MOLECULAR DOCKING STUDY OF CDK2 INHIBITORS WITH POTENT CYTOTOXIC ACTIVITY AGAINST HCT116 COLORECTAL CANCER CELL LINE**

**SUPPLEMENTARY TABLES**

**Supplementary Table S1:** External validation of built model

| Name | Yexp. | Ypred. | (Yexp - Ypred) | (Yexp - Ypred)^2^ | (Yexp - ${\bar{\boldsymbol{Y}}}_{\boldsymbol{train}}$) | (Yexp - ${\bar{\boldsymbol{Y}}}_{\boldsymbol{train}}$)^2^ |
| --- | --- | --- | --- | --- | --- | --- |
| 5 | 4.1896 | 4.13347 | 0.05613 | 0.00315 | 0.1860 | 0.03459 |
| 21 | 5.1669 | 5.08665 | 0.08025 | 0.00644 | -0.7913 | 0.62617 |
| 22 | 4.6492 | 4.98419 | -0.33499 | 0.11222 | -0.2736 | 0.07486 |
| 20 | 5.3125 | 5.25704 | 0.05546 | 0.00308 | -0.9369 | 0.87780 |
| 28 | 4.1323 | 4.40987 | -0.27757 | 0.07705 | 0.2433 | 0.05919 |
| 19 | 4.8633 | 5.28204 | -0.41874 | 0.17534 | -0.4877 | 0.23786 |
| 33 | 4.4616 | 4.5587 | -0.09710 | 0.00943 | -0.0860 | 0.00740 |
| 35 | 4.0819 | 4.20053 | -0.11863 | 0.01407 | 0.2937 | 0.08625 |
| 36 | 4.543 | 4.43358 | 0.10942 | 0.01197 | -0.1674 | 0.02803 |
| 2 | 4.4131 | 4.76675 | -0.35365 | 0.12507 | -0.0375 | 0.00141 |
| 4 | 4.2771 | 4.46094 | -0.18384 | 0.03380 | 0.0985 | 0.00970 |
| $\sum\left( Y_{exp}-Y_{pred} \right)^{2}=0.571616$ | | | | $\sum\left( Y_{exp}-\bar{Y}_{train} \right)^{2}=2.043255$ | | |
| ${R^{2}}_{ext}=1-\frac{\sum\left( Y_{exp}-Y_{pred} \right)^{2}}{\sum\left( Y_{exp}-\bar{Y}_{train} \right)^{2}}=1-\frac{0.571616}{2.043255}=0.720242$ | | | | | | |

**Supplementary Table S2:** Experimental, predicted and residual cytotoxic activity of training set compounds

| **Name** | **nS** | **GATS5s** | **VR1_Dze** | **ETA_dBetaP** | **L3i** | **Yexp** | **Ypred.** | **Residual** |
| --- | --- | --- | --- | --- | --- | --- | --- | --- |
| 6 | 1 | 1.009115 | 271.4019 | 0.25 | 0.688505 | 4.0124 | 4.1584 | -0.1460 |
| 7 | 1 | 1.009096 | 828.7128 | 0.24038 | 0.711466 | 4.0374 | 4.1747 | -0.1373 |
| 8 | 1 | 0.957279 | 947.2641 | 0.22115 | 0.697497 | 4.1668 | 4.2278 | -0.0610 |
| 9 | 1 | 0.936794 | 371.4335 | 0.25 | 1.034905 | 4.2637 | 4.4955 | -0.2318 |
| 10 | 1 | 0.883835 | 340.4138 | 0.21429 | 1.26269 | 4.3478 | 4.2634 | 0.0844 |
| 11 | 1 | 0.910625 | 306.6751 | 0.21429 | 0.671586 | 4.107 | 4.2197 | -0.1127 |
| 12 | 1 | 0.910388 | 409.0598 | 0.18966 | 0.696852 | 4.1121 | 3.9278 | 0.1843 |
| 13 | 0 | 0.969513 | 515.5377 | 0.25714 | 0.759967 | 4.2945 | 4.6284 | -0.3339 |
| 14 | 0 | 0.968335 | 582.5951 | 0.25 | 1.326924 | 4.4947 | 4.4619 | 0.0328 |
| 15 | 0 | 0.971453 | 536.7401 | 0.24306 | 0.746887 | 4.4344 | 4.4460 | -0.0116 |
| 16 | 1 | 1.032315 | 505.9698 | 0.27143 | 1.925479 | 4.2435 | 4.1589 | 0.0846 |
| 17 | 1 | 1.031429 | 600.4423 | 0.26389 | 2.035329 | 4.0759 | 4.0722 | 0.0037 |
| 18 | 1 | 1.034529 | 546.5569 | 0.25694 | 1.908918 | 4.0176 | 3.9755 | 0.0421 |
| 1 | 0 | 0.918138 | 263.8605 | 0.23 | 0.6808 | 4.8368 | 4.4943 | 0.3425 |
| 24 | 1 | 0.947046 | 463.6101 | 0.27027 | 0.751271 | 5.2269 | 4.7751 | 0.4518 |
| 23 | 1 | 0.940682 | 451.396 | 0.27703 | 0.720859 | 4.9423 | 4.8964 | 0.0459 |
| 25 | 1 | 0.828802 | 866.936 | 0.20395 | 0.245136 | 4.7605 | 4.7258 | 0.0347 |
| 26 | 1 | 0.82683 | 526.5713 | 0.19872 | 0.247889 | 4.7828 | 4.5811 | 0.2017 |
| 27 | 1 | 0.834602 | 516.8281 | 0.19231 | 0.247996 | 4.3773 | 4.4566 | -0.0793 |
| 29 | 2 | 0.904877 | 531.4052 | 0.21154 | 0.252223 | 4.0564 | 4.2197 | -0.1633 |
| 30 | 2 | 0.912813 | 520.417 | 0.20513 | 0.25058 | 4.2054 | 4.0944 | 0.1110 |
| 31 | 0 | 0.823471 | 309.7021 | 0.17241 | 1.205518 | 4.0696 | 4.1647 | -0.0951 |
| 32 | 1 | 0.882264 | 310.0317 | 0.18966 | 1.315623 | 4.0325 | 3.9394 | 0.0931 |
| 34 | 1 | 0.829579 | 303.0614 | 0.22581 | 1.013891 | 4.3694 | 4.7231 | -0.3537 |
| 3 | 0 | 0.868039 | 3062.337 | 0.20192 | 0.694892 | 5.1221 | 5.1091 | 0.0130 |

**Key:** Yexp. = Experimental activity, Ypred. = Predicted activity

**Supplementary Table S3:** Experimental, predicted and residual cytotoxic activity of test set compounds

| **Name** | **nS** | **GATS5s** | **VR1_Dze** | **ETA_dBetaP** | **L3i** | **Yexp.** | **Ypred.** | **Residual** |
| --- | --- | --- | --- | --- | --- | --- | --- | --- |
| 5 | 0 | 0.850106 | 374.6296573 | 0.17241 | 0.693323 | 4.1896 | 4.1335 | 0.0561 |
| 21 | 0 | 0.866669 | 466.6174147 | 0.25676 | 1.063351 | 5.1669 | 5.0867 | 0.0802 |
| 22 | 1 | 0.941306 | 418.9147797 | 0.28472 | 0.714887 | 4.6492 | 4.9842 | -0.3350 |
| 20 | 0 | 0.860432 | 453.4243141 | 0.26351 | 0.744172 | 5.3125 | 5.2570 | 0.0555 |
| 28 | 2 | 0.906508 | 1026.520651 | 0.21711 | 0.251746 | 4.1323 | 4.4099 | -0.2776 |
| 19 | 0 | 0.861441 | 417.9060023 | 0.27083 | 1.057132 | 4.8633 | 5.2820 | -0.4187 |
| 33 | 1 | 0.825235 | 642.0183564 | 0.22581 | 2.591206 | 4.4616 | 4.5587 | -0.0971 |
| 35 | 2 | 0.90112 | 484.1989697 | 0.24194 | 2.644235 | 4.0819 | 4.2005 | -0.1186 |
| 36 | 2 | 0.905515 | 306.0114078 | 0.24194 | 0.907709 | 4.543 | 4.4336 | 0.1094 |
| 2 | 0 | 0.917548 | 1766.59838 | 0.22115 | 0.700719 | 4.4131 | 4.7668 | -0.3537 |
| 4 | 0 | 0.825444 | 331.8353834 | 0.19643 | 1.243343 | 4.2771 | 4.4609 | -0.1838 |

**Key:** Yexp. = Experimental activity, Ypred. = Predicted activity

**Supplementary Table S4:** Molecular descriptors and predicted cytotoxic activity of designed compounds

| **Name** | **nS** | **GATS5s** | **VR1_Dze** | **ETA_dBetaP** | **L3i** | **pIC50** | **IC50** |
| --- | --- | --- | --- | --- | --- | --- | --- |
| a | 0 | 0.863572 | 467.5689 | 0.26351 | 1.242342 | 5.1581 | 6.94896 |
| b | 0 | 0.854994 | 531.0013 | 0.26923 | 1.194585 | 5.2999 | 5.01310 |
| c | 0 | 0.803045 | 451.8266 | 0.25676 | 1.303048 | 5.3681 | 4.28427 |
| d | 0 | 0.861465 | 452.8083 | 0.25676 | 1.10766 | 5.1022 | 7.90403 |
| e | 0 | 0.77958 | 556.5051 | 0.24342 | 0.957495 | 5.4049 | 3.93616 |
| f | 0 | 0.86149 | 565.2467 | 0.24342 | 1.071112 | 4.9666 | 10.7985 |
| g | 0 | 0.86259 | 652.6425 | 0.25658 | 1.24026 | 5.1224 | 7.54401 |
| h | 0 | 0.680404 | 622.0704 | 0.24342 | 1.329147 | 5.8668 | 1.35897 |
| i | 0 | 0.866722 | 640.0991 | 0.24342 | 1.158441 | 4.9438 | 11.3808 |
| j | 0 | 0.815846 | 608.9728 | 0.26923 | 1.442581 | 5.4780 | 3.32652 |
| k | 0 | 0.859447 | 571.0336 | 0.25658 | 1.033198 | 5.1535 | 7.02236 |
| l | 0 | 0.757005 | 556.8743 | 0.26786 | 1.524886 | 5.7351 | 1.84027 |
| 20 | 0 | 0.860432 | 453.4243 | 0.26351 | 0.744172 | 5.2570 | 5.53296 |
